# Association between Race and Cancer-Related Mortality among Patients with Colorectal Cancer in the United States: A Retrospective Cohort Study

**DOI:** 10.3390/ijerph16020240

**Published:** 2019-01-16

**Authors:** Sayaf H. Alshareef, Nasser A. Alsobaie, Salman A. Aldeheshi, Sultan T. Alturki, Juan Carlos Zevallos, Noël C. Barengo

**Affiliations:** 1 College of Medicine, Imam Muhammad Ibn Saud Islamic University, Riyadh 13317, Saudi Arabia; nasseralsobaie@gmail.com (N.A.A.); salmandeh@gmail.com (S.A.A.); sultantalturki@gmail.com (S.T.A.); 2 Department of Medical and Population Health Sciences Research, Herbert Wertheim College of Medicine, Florida International University, Miami, FL 33199, USA; juzevall@fiu.edu (J.C.Z.); nbarengo@fiu.edu (N.C.B.)

**Keywords:** race, blacks, colorectal cancer, death

## Abstract

Colorectal cancer (CRC) is the third most common cause of mortality in the United States (US). Differences in CRC mortality according to race have been extensively studied; however, much more understanding with regard to tumor characteristics’ effect on mortality is needed. The objective was to investigate the association between race and mortality among CRC patients in the US during 2007–2014. A retrospective cohort study using data from the Surveillance, Epidemiology, and End Results (SEER) Program, which collects cancer statistics through selected population-based cancer registries during in the US, was conducted. The outcome variable was CRC-related mortality in adult patients (≥18 years old) during 2007–2014. The independent variable was race of white, black, Asian/Pacific Islander (API), and American Indian/Alaska Native (others). The covariates were, age, sex, marital status, health insurance, tumor stage at diagnosis, and tumor size and grade. Bivariate analysis was performed to identify possible confounders (chi-square tests). Unadjusted and adjusted logistic regression models were used to study the association between race and CRC-specific mortality. The final number of participants consisted of 70,392 patients. Blacks had a 32% higher risk of death compared to whites (adjusted odds ratio (OR) 1.32; 95% confidence interval (CI) 1.22–1.43). Corresponding OR for others were 1.41 (95% CI 1.10–1.84). API had nonsignificant adjusted odds of mortality compared to whites (0.95; 95% CI 0.87–1.03). In conclusion, we observed a significant increased risk of mortality in black and American Indian/Alaska Native patients with CRC compared to white patients.

## 1. Introduction

Colorectal cancer (CRC) is the third most commonly diagnosed cancer in the United States (US) among all races and both sexes [1,2]. It has been estimated that 100,000 new cases of CRC are diagnosed each year [1]. The incidence and mortality of colorectal cancer has been decreasing during the last two decades; however, a divergence in mortality still exists between different races [3]. Research showed that whites have a higher survival rate in colon cancer than blacks. The lack of providing the appropriate healthcare burdens cancer screening programs and delays tumor diagnosis to advanced stages [4]. Therefore, blacks are more likely to present with advanced tumors at the time of diagnosis compared to other races [4]. Consequently, blacks have higher mortality rates of colorectal cancer [5,6]. Even though tumor characteristics have been extensively studied, tumor size is yet to be fully addressed as a confounding factor for CRC-related mortality. Furthermore, previous studies did not control for tumor size in their analysis, as tumor size at diagnosis may differ between races.

Differences in mortality among people of different race may be due to socioeconomic status, age, sex, stage of tumor at time of diagnosis, and colorectal cancer screening programs [7,8,9,10]. However, even after controlling for some of these characteristics, differences in survival persisted [11]. 

Some studies reported that the stage of tumor at the time of diagnosis was the major factor explaining the mortality disparity among different races in the US [12]. 

A recent study revealed that when races were compared, assuming an equal access to health care, the race–survival disparity disappeared in patients above 50 years of age [7]. Young black Americans (<50 years old) have a higher risk of death than their white American counterparts; furthermore, authors observed an increased risk of death in only black men compared to white, with no difference among women [13]. In another study which investigated the differences in colorectal cancer by race and insurance, authors concluded that patients with no insurance or those who have Medicaid have lower survival rates than those with private insurance. However, there is no difference in the receipt of treatment for colorectal cancer between whites and blacks [14]. 

The aim of this study was to investigate the association between race and mortality among patients with CRC in the US during 2007–2014.

## 2. Materials and Methods 

### 2.1. Study Design and Population

The design of this secondary data analysis was a retrospective cohort study using the available data from the Surveillance, Epidemiology, and End Results (SEER) Program of the National Cancer Institute [15]. SEER collects mortality data among patients with cancer through selected population-based cancer registries covering approximately 28 percent of the US population in 17 states. 

The inclusion criteria for the study participants were adults (≥18 years old) diagnosed with primary colorectal cancer using the international classification of diseases (ICD-10) codes for colon and rectal cancers (C180, C182, C183, C184, C185, C186, C187, C189, C199, and C209) between 2007 and 2014 in the US. People with metastatic colon cancer (cancers that developed metastasis in the colon or rectum but were not primary colorectal cancers) or missing data in any variable were excluded. 

The main outcome of the study was CRC-specific mortality. Race, the main exposure variable, was included the following racial/ethnic categories: Whites, blacks, Asians/Pacific Islanders, American Indians, and Alaska Natives. The covariates included in the study were sex, age at diagnosis, marital status, insurance status, diagnosis stage, tumor grade, and tumor size. Age at diagnosis was categorized into ≥18–50, 51–70, 71–80, and >80 years-old groups. Marital status was registered as married, unmarried (single, divorced, widowed separated, unmarried or domestic partner) or unknown. Health insurance was dichotomized into insured (including Medicaid) and uninsured, as per SEER insurance code. The stage of tumor was classified according to SEER Summary stage 2000 (1998+) as in situ, localized, regional or distant. Tumor grade was categorized into four different groups: (i) Differentiated; (ii) moderately differentiated; (iii); poorly differentiated; and (iv) undifferentiated. Tumor size was reported in SEER as exact size in millimeters. For our analysis, tumor size was dichotomized into less or above 40 mm according to the median tumor size of the study participants. Information on health insurance was not available prior to 2007 in SEER; the time span for this study was 2007–2014.

### 2.2. Statistical Analysis

Stata V. 14 (StataCorp LLC, College Station, TX, USA) was used to conduct all analyses. Exploratory analysis was carried out by examining frequency distribution. A Chi-square test was used to test bivariate associations between potential confounders according to race and mortality, respectively. The covariates of age, sex, age at diagnosis, marital status, insurance status, stage at diagnosis, and tumor grade and size were found to be differently distributed according to the exposure and outcome variables. Thus, the mathematical models were adjusted for these eight variables. Binary logistic regression was used to estimate unadjusted and adjusted odds ratios (OR) and 95% confidence intervals (CI) between race and mortality. The main outcome variable was CRC-specific mortality and the main exposure variable was race. The Hosmer Lemeshow test was applied to test for the goodness-of-fit of the statistical model. A *p*-value of less than 0.05 was considered statistically significant. 

### 2.3. Ethical Considerations

Ethical approval was waived, since the analysis was considered nonhuman subjects research by the Florida International University Health Science Institutional Review Board.

### 2.4. Data Availability

The Surveillance, Epidemiology, and End Results (SEER) data used to support the findings of this study were supplied by the National Cancer Institute under license and so cannot be made freely available. Requests for access to these data should be made to the National Cancer Institute, https://seer.cancer.gov [15].

## 3. Results

The total number of participants in this study was 85,796 and after applying exclusion criteria, the eligible number of participants was 70,392. Table 1 presents the characteristics of the study population according to race. A statistically significant difference was observed between the distributions of sex and tumor size according to race. The prevalence of American Indians/Alaskan natives diagnosed with colorectal cancer between 18 and 50 years of age was 21.8%. Approximately half of (54%) of blacks were diagnosed at 51–70 years of age, and most of the whites were diagnosed at a late age (*p* < 0.001). American Indians/Alaskan natives had a higher percentage of grade I (11.2%) compared with other race groups (*p* < 0.001). In addition, Asians/Pacific Islanders had the highest percentage of Grade II tumor (77.7%) and whites had the highest frequency of grade III and grade IV tumor (16.9% and 2.7%, respectively) (*p* < 0.001). About 5.7% of blacks were uninsured, which was higher than the corresponding prevalence among whites, Asians/Pacific islanders, and American Indians (2.3%, 2.2%, and 1.5%, respectively). Furthermore, blacks had a higher prevalence of being unmarried (63.3%) than other races (*p* < 0.001). Diagnosis stage showed statistically significant differences among colorectal cancer patients. 

Table 2 presents the characteristics of colorectal cancer patients according to cause-specific mortality. Black people had a higher mortality (30.3%) in comparison to other races (*p* < 0.001). Female colorectal cancer patients died more often than male patients (*p* = 0.035). Patients with a tumor size ≥41 mm had a higher mortality compared to those with a tumor size <41 mm (28.9% vs 15.9%; *p*-value < 0.001). Furthermore, statistically significant differences in mortality were observed according to marital status, stage at diagnosis, age groups, and tumor grade as well (*p*-values < 0.001). 

Table 3 shows the unadjusted and adjusted associations between race and cancer-specific death. Blacks had the highest odds for colorectal cancer mortality with a statistically significant 32% increased odds compared with whites (OR = 1.32; 95% CI 1.22–1.43). The corresponding OR for American Indians/Alaskan Natives was 1.41 (95% CI 1.10–1.84). The unadjusted OR for mortality in API showed a lower risk compared with whites (OR = 0.88, 95% CI 0.83–0.93). However, after adjusting for the covariates (sex, age at diagnosis, tumor size, tumor grade, insurance status, marital status, and diagnosis stage) the decrease in odds became statistically nonsignificant (OR = 0.95, 95% CI 0.87–1.03). It can be seen that the confidence intervals of the adjusted odds of blacks is included within the confidence limits of American Indians/Alaskan Natives, indicating that there are no differences in the odds of mortality between these two groups. Unmarried (single, divorced, widowed, and separated) patients had a 54% increase in the odds of mortality compared to married patients (OR = 1.54, 95% CI 1.49–1.60). Additionally, the odds of mortality in the adjusted logistic regression models in uninsured CRC patients was 51% higher than in insured patients (OR = 1.51, 95% CI 1.37–1.67).

## 4. Discussion

Our study found disparities between races regarding colorectal cancer-specific mortality. Blacks, American Indians, and Alaskan Natives had a higher mortality compared with whites. 

Previous studies mainly compared blacks or African Americans to whites [16,17,18,19]. One study included Asian Americans and Hispanics but did not include native Americans [18]. However, Ward et al. included native Americans along with “other” races (African American, white, non-Hispanic Whites, and Asian/Pacific Islander) [16]. Most of the studies used the SEER database with years ranging from 1975–2000 and 1992–2000 [16], 1974–1976, 1983–1985, 1995–2001 [17], 1988–2006 [18], and one study in Delaware, US, investigated trends in CRC screening, incidence, and mortality in 2001 and 2009 [19]. Our study is consistent with the previous studies that blacks had higher CRC- related mortality compared to whites. Moreover, Aizer et al. reported that African Americans were more likely to be diagnosed with advanced CRC stages and sought medical therapy less often [18]. Nevertheless, racial disparity in CRC-related mortality persisted independently of CRC tumor stage and treatment [18].

In a different study, Alexander et al. reviewed epidemiologic studies from the Veterans Affairs health care system database among other databases and registries and found that even in a setting where equal access to care and treatment existed, as in Veteran Affairs hospitals, the same finding persisted [17].

In the US, Delaware created a comprehensive state-wide CRC screening program in 2002 that included coverage for screening and treatment [19]. Grubbs et al. studied trends in CRC screening, incidence, and mortality in 2001 and 2009, and found that even though screening and incidence rates disparities were eliminated in 2009, blacks still had higher mortality rates compared to whites [19].

The disparity in mortality can be explained by various mechanisms, such as insurance, which was demonstrated by Tawk et al. [14] to have a protective effect. Genetic and biological predisposition are also possible mechanisms, Agrawal et al. [20] found in their study that microsatellite instability plays a role in CRC course and prognosis. Duray et al. [21] reported that aged people have CRC with poorer prognosis and that could be attributed to weakness and instability of the immune system. Psychosocial factors are also suggested to have a role, as Li et al. [22] found that unmarried people have a worse outcome compared to married people.

The present study shows that whites tended to be diagnosed with colorectal cancer late in their lives. By contrast, American Indians/Alaskan Natives and blacks tended to be diagnosed with colorectal cancer in younger ages. Literature reported that American Indians/Alaskan Natives had the tendency to be diagnosed earlier [23]. This might be due to other health risk factors, as American Indians/Alaska Natives were found to have heavy alcohol drinking and smoking habits in comparison to whites [24,25]. Heavy alcohol consumption and smoking have been associated with increased risk of colorectal cancer [26,27,28]. A study reported that African Americans have the tendency to be diagnosed with colorectal cancer earlier than others [29]. It is unclear why blacks had the tendency to present with the disease earlier than others, but it could be explained by multiple factors, such as genetic and biological predisposition or the current guidelines recommending that cancer screening should only be started at age of 50 years, which might be late for them [20]. Additionally, studies reported that there is a higher proportion of right-sided colon cancer among African Americans, which might obscure screening tests (flexible sigmoidoscopy) and prevent early diagnosis [30,31,32,33]. CRC-related mortality is directly associated with increasing age at time of diagnosis and that was consistent with the literature [22]. That could be explained by weakened immune response and increased oxidative stress [21,34]. Unmarried patients were more likely to die of colorectal cancer and had lower survival rates and this was consistent with Li et al.’s study; this may be attributed to a lack of psychosocial support [35,36,37]. Our study also shows that blacks were more likely to be unmarried, compared to other races. Uninsured patients had increased odds of mortality (OR 1.21, 95% CI 1.03–1.41) compared to those insured, which was consistent with Tawk et al.’s study. Lin et al. reported that blacks were less likely to be insured, which is consistent with the findings in our study. To our knowledge, there has not been a study that investigated the association of colorectal cancer-related mortality and tumor size. We found that the larger the tumor size, the higher the risk of cancer-related mortality. Even though we found an association between tumor size and cancer-related mortality, our results need to be interpreted with caution as we used a median value of tumor size and used a dichotomized variable for the statistical modeling. Some studies about the association between tumor size and prognosis have been published [38,39]. In general, tumor size seems to be inversely associated with survival [38]. However, studies using SEER data also reported an unfavorable effect of small tumor size on survival in stage-II [38] and T4b [39] colon cancers. Our study also reported that blacks and American Indians/Alaskan Natives had larger tumor size. This might add to the fact that both races had increased odds of cancer-related mortality in our study (OR 1.32, 95% CI 1.22–1.43), (OR 1.41, 95% CI 1.10–1.84), respectively. It has been shown that both advanced tumor grades and stages of colorectal cancers are associated with increased mortality [13]. This was generally supported by our study as well, revealing that both grade and stage affected the odds of mortality when adjusting for race, even though higher grades were found among whites that have less mortality. On the other hand, blacks were more likely be diagnosed with distant colorectal cancer stage, consistent with other studies [40,41,42]. The independent effect of grade and stage may cancel each other out in the adjusted analysis. Another hypothesis may be that blacks are less likely to undergo screening tests and receiving adequate treatment [43].

Naturally, our study had some limitations: The SEER database does not include data about socioeconomic status, income, lifestyle, education, comorbidities, and use of screening test or chemotherapy treatment. Additionally, it only has data from 17 US states’ registries. Asians and Pacific islanders were grouped as one entity, but they may have different genetic and risk factors. Furthermore, genetic and biological data are not incorporated in the database. 

## 5. Conclusions

In conclusion, our study reveals blacks had the highest CRC-related mortality among all races included in the study compared to whites. Demographic features and tumor characteristics may play a role in the disparity of CRC-related mortality. The establishment of early screening for high-risk groups might overcome those disparities. We also recommend awareness programs targeted at those with a higher risk of CRC to encourage early screening and adherence to screening programs. Additionally, further research is needed to investigate the reasons for delayed screening in at-risk groups. Other studies focusing on behavior, lifestyle, knowledge, and attitude should be done to further explain the disparities in the outcome. Due to the higher proportion of right-sided tumors, diagnostic tools should put emphasis on covering the whole colon.

## Figures and Tables

**Table 1:** Characteristics of colorectal cancer patients according race in the US during 2007 and 2014.

Characteristics	Race				*p*-Value ^3^
	White (*n* = 54,125)	Black (*n* = 8020)	API ^1^ (*n* = 7627)	Other ^2^ (*n* = 593)	
	*n* (%)	*n* (%)	*n* (%)	*n* (%)	
Sex					<0.001
Male	28,382 (52.4)	3959 (49.4)	4178 (54.8)	303 (53.2)	
Female	25,743 (47.6)	4061 (50.6)	3449 (45.2)	290 (46.8)	
Age at diagnosis					<0.001
≥18–50 years	7174 (13.3)	1371 (17.1)	1158 (15.2)	135 (21.8)	
51–70 years	24,030 (44.4)	4357 (54.3)	3762 (49.3)	322 (51.9)	
71–80 years	12,283 (22.7)	1411 (17.6)	1574 (20.6)	117 (18.9)	
>80 years	10,638 (19.7)	881 (11)	1133 (14.9)	46 (7.4)	
Marital status					<0.001
Unmarried	21,544 (42.2)	4720 (63.3)	2580 (36.4)	282 (51.9)	
Married	29,533 (57.8)	2733 (36.7)	4511 (63.6)	261 (48.1)	
Insurance status					<0.001
Uninsured	1175 (2.3)	446 (5.7)	163 (2.2)	9 (1.5)	
Insured	50,960 (97.8)	7349 (94.3)	7113 (97.8)	584 (98.5)	
Diagnosis stage					<0.001
In situ	1705 (3.2)	242 (3.1)	292 (3.9)	11 (1.8)	
Localized	20,798 (39.3)	2722 (34.8)	2854 (38.4)	214 (35.3)	
Regional	20,039 (37.9)	2779 (35.5)	2882 (38.7)	242 (39.9)	
Distant	10,393 (19.6)	2083 (26.6)	1413 (19)	140 (23.1)	
Tumor grade					<0.001
Grade I	4396 (9.5)	564 (8.6)	475 (7.2)	60 (11.2)	
Grade II	32,988 (71)	5029 (76.3)	5116 (77.7)	396 (73.9)	
Grade III	7850 (16.9)	900 (13.7)	888 (13.5)	71 (13.3)	
Grade IV	1258 (2.7)	99 (1.5)	107 (1.6)	9 (1.7)	
Tumor size					<0.001
≤40 mm	21,564 (50.2)	2979 (47.2)	3278 (53.2)	200 (42.8)	
≥41 mm	21,374 (49.8)	3329 (52.8)	2882 (46.8)	267 (57.2)	

^1^ Asian or Pacific Islanders; ^2^ American Indians and Alaska Natives; ^3^ Chi-square test.

**Table 2:** Characteristics of colorectal cancer patients according to colorectal cancer specific mortality in the US during 2007 and 2014.

Characteristics	Alive or Dead Due to Other Cause	Dead	*p*-Value ^1^
	*n* (%)	*n* (%)	
Race			<0.001
White	40,928 (75.6)	13,197 (24.4)	
Black	5587 (69.7)	2433 (30.3)	
API ^2^	5946 (78)	1681 (22)	
Other ^3^	441 (71.1)	179 (28.9)	
Sex			0.035
Male	27,814 (75.5)	9035 (24.5)	
Female	25,088 (74.8)	8455 (25.2)	
Age at diagnosis			<0.001
≥18–50	7735 (78.6)	2103 (21.4)	
51–70	25,529 (78.6)	6942 (21.4)	
71–80	11,481 (74.6)	3904 (25.4)	
>80	8157 (64.2)	4541 (35.8)	
Marital status			<0.001
Unmarried	20,493 (70.4)	8633 (29.6)	
Married	29,086 (78.5)	7952 (21.5)	
Insurance Status			<0.001
Uninsured	1200 (66.9)	593 (33.1)	
Insured	49,768 (75.4)	16,238 (24.6)	
Stage at diagnosis			<0.001
In situ	2203 (97.9)	47 (2.1)	
Localized	24,644 (92.7)	1944 (7.3)	
Regional	20,658 (79.6)	5284 (20.4)	
Distant	4555 (32.5)	9474 (67.5)	
Tumor grade			<0.001
Grade I	4690 (85.4)	805 (14.7)	
Grade II	34,363 (78.9)	9166 (21.1)	
Grade III	6076 (62.6)	3633 (37.4)	
Grade IV	994 (67.5)	479 (32.5)	
Tumor size			<0.001
≤40 mm	23,571 (84.1)	4450 (15.9)	
≥41 mm	19,812 (71.1)	8040 (28.9)	

^1^ Chi-square test; ^2^ Asian or Pacific Islanders; ^3^ American Indians and Alaska Natives.

**Table 3:** Unadjusted and adjusted associations of the logistic regression models between race and cause specific death in patients with colorectal cancer in the US during 2007–2014.

Characteristics	Unadjusted	Adjusted ^1^
	OR ^2^ (95% CI ^3^)	OR (95% CI)
Race		
White	Ref ^4^	Ref
Black	1.35 (1.28–1.42)	1.32 (1.22–1.43)
API ^5^	0.88 (0.83–0.93)	0.95 (0.87–1.03)
Other ^6^	1.26 (1.06–1.50)	1.41 (1.10–1.84)
Sex		
Male	Ref	Ref
Female	1.04 (1.00–1.07)	0.87 (0.83–0.92)
Age at diagnosis		
≥18–50	Ref	Ref
51–70	1.00 (0.95–1.06)	1.24 (1.15–1.35)
71–80	1.25 (1.18–1.33)	1.90 (1.74–2.07)
>80	2.05 (1.93–2.17)	3.17 (2.90–3.47)
Marital status		
Married	Ref	Ref
Unmarried	1.54 (1.49–1.60)	1.27 (1.20–1.34)
Insurance status		
Insured	Ref	Ref
Uninsured	1.51 (1.37–1.67)	1.21 (1.03–1.41)
Tumor grade		
Grade I	Ref	Ref
Grade II	1.55 (1.44–1.68)	1.02 (0.92–1.14)
Grade III	3.48 (3.20–3.79)	1.74 (1.55–1.95)
Grade IV	2.81 (2.46–3.20)	1.47 (1.24–1.74)
Diagnosis stage		
Localized	Ref	Ref
In situ	0.27 (0.20–0.36)	0.30 (0.11–0.82)
Regional	3.24 (3.07–3.43)	3.19 (2.98–3.42)
Distant	26.37 (24.88–27.95)	23.55 (21.77–25.48)
Tumor size		
≤40 mm	Ref	Ref
≥41 mm	2.15 (2.06–2.24)	1.21 (1.15–1.27)

^1^ Adjusted for the covariates: Sex, age at diagnosis, tumor size, tumor grade, insurance status, marital status, and diagnosis stage; ^2^ Odds ratio; ^3^ Confidence interval; ^4^ Reference group; ^5^ Asian or Pacific Islanders; ^6^ American Indians and Alaska Natives.
